# Soluble POSTN is a novel biomarker complementing CA153 and CEA for breast cancer diagnosis and metastasis prediction

**DOI:** 10.1186/s12885-022-09864-y

**Published:** 2022-07-12

**Authors:** Li Jia, Guanhua Li, Na Ma, Aimin Zhang, Yunli Zhou, Li Ren, Dong Dong

**Affiliations:** 1 Department of Laboratory, Tianjin’s Clinical Research Center for Cancer; Key Laboratory of Breast Cancer Prevention and Therapy, Tianjin Medical University, Ministry of Education; Key Laboratory of Cancer Prevention and Therapy, Tianjin, Tianjin Medical University Cancer Institute and Hospital, National Clinical Research Center for Cancer, Tianjin, 300060 People’s Republic of China; 2 Department of Anesthesiology, Chinese People’s Liberation Army Rocket Force Characteristic Medical Center, Beijing, 100088 People’s Republic of China; 3 Cancer Biobank, Tianjin’s Clinical Research Center for Cancer; Key Laboratory of Breast Cancer Prevention and Therapy, Tianjin Medical University, Ministry of Education; Key Laboratory of Cancer Prevention and Therapy, Tianjin, Tianjin Medical University Cancer Institute and Hospital, National Clinical Research Center for Cancer, Tianjin, 300060 People’s Republic of China

**Keywords:** Biomarker, Breast cancer, Early diagnosis, Metastasis prediction, Prognosis, Soluble POSTN

## Abstract

**Background:**

Breast cancer (BCa) is the leading cause of cancer deaths among women. Reliable biomarkers for early diagnosis and metastasis prediction are essential to improve the prognosis of BCa. This study aimed to evaluate serum periostin (POSTN) as a novel biomarker complementing CA153 (carbohydrate antigen 153) and CEA (carcinoembryonic antigen) for BCa diagnosis and metastasis prediction.

**Methods:**

To assess the potential of soluble POSTN as a circulating biomarker, 242 participants, including 173 patients with different stages of BCa and 69 healthy individuals, were enrolled in this study. Soluble POSTN, together with CA153 and CEA, were determined in serum by enzyme linked immunosorbent assay (ELISA) or electrochemiluminescence immunoassays.

**Results:**

Serum POSTN levels in locoregional BCa patients were significantly higher than that in healthy controls. Receiver operating curve (ROC) analysis revealed that, to distinguish health controls from locoregional BCa, POSTN was observed with the highest AUC **(**area under curve**)** (AUC_POSTN_ = 0.72 [0.65 – 0.79], AUC_CA153_ = 0.57 [0.49 – 0.64], AUC_CEA_ = 0.62 [0.55 – 0.69]), and both CA153 and CEA were observed with significantly improved AUCs by combination with POSTN (AUC_POSTN + CA153_ = 0.74 [0.67 – 0.80], *P* < 0.001; AUC_POSTN + CEA_ = 0.77 [0.70 – 0.82], *P* < 0.001). Moreover, the performances of the POSTN were comparable with that of CA153 in predicting distant metastasis of BCa (AUC_POSTN_ = 0.78 [0.71 – 0.84], AUC_CA153_ = 0.82 [0.76 – 0.88]). Kaplan–Meier analysis indicated that elevated serum POSTN was associated with poor overall survival and progression-free survival.

**Conclusions:**

This study suggested that soluble POSTN is a promising potential biomarker for diagnosis and metastasis prediction of BCa.

**Supplementary Information:**

The online version contains supplementary material available at 10.1186/s12885-022-09864-y.

## Background

Breast cancer (BCa) is a leading cause of cancer deaths among women and accounts for approximately 30% all new female cancers, leading to a huge global disease burden [1]. Thanks to the availability of more effective systemic treatment regimens, the prognosis of patients with early-stage disease has been significantly improved in recent years [1]. However, due to with almost no symptoms in the early stages, about 5–10% of newly diagnosed BCa patients have already developed metastatic disease [2]. In addition, about 20–30% of early BCa patients will have metastatic recurrence, which is a major clinical manifestation of BCa and the main cause of BCa-related deaths [3, 4]. According to the stage at diagnosis, the 5-year relative survival rate for women with BCa varied from 99% for local cancer to 84% for regional cancer and 23% for disease in the metastasis phase [5]. The survival rate are highly varied between patients with locoregional BCa and distant BCa. Overall, early detection and metastasis prediction are essential to improve the prognosis of BCa.

Breast imaging modalities, such as magnetic resonance imaging (MRI) and computed tomographic (CT), are clinically used to evaluate the dynamics of BCa lesions [6]. But their main disadvantages lay on the high cost, long-time of scanning and possible exposure to contrast agents. Another major concerns with these tests is radiation, which could act as a contributor to the onset of cancer [7, 8]. Therefore, none of these procedures have proven to be effective screening tools for patients in both general and high-risk populations. On the contrary, convenient and non-invasive blood tests have been widely accepted and are routinely used to determine biomarkers. Carcinoembryonic antigen (CEA) and carbohydrate antigen 153 (CA153) are the most commonly used blood markers in BCa management. The fact that CEA and/or CA153 are not elevated in the serum of a certain proportion of BCa patients suggests that they are not sensitive enough for disease screening [9]. Taken together, there is an urgent need to explore other novel and reliable non-invasive biomarkers. And in the past decades, much attention and research work have been focused on the development of efficient biomarkers to complement the current clinical approaches for early diagnosis of BCa [10].

The aim of this work was to explore novel circulating protein biomarkers for BCa. We first identified the top differentially expressed genes, coding secreted plasma proteins, between BCa and all other normal human tissues with bioinformatics-based methods. And then, we validated the clinical significance of the candidate biomarker in diagnosis, metastasis prediction, and prognosis of BCa.

## Methods

### Patients and specimens for validation study

This study was conducted at Tianjin Medical University Cancer Institute and Hospital between January 2016 and February 2020. All procedures performed in studies involving human participants were approved by the Research Ethics Committee of Tianjin Medical University Cancer Institute and Hospital, and in accordance with the 1964 Helsinki Declaration ethical standards. All patient samples were collected following written informed consent, and the study has been approved by the local Ethical Board. All patients with BCa were confirmed by pathologic examination.

For validation study, 242 participants, including 173 patients with different stages of BCa, and 69 healthy individuals were enrolled. Healthy controls without BCa were determined by mammogram or breast ultrasound. Although not an age-matched study, the median age of healthy controls was controlled to be similar to BCa patients. Serum samples were harvested before surgery or treatment and were collected, aliquoted, and snap frozen at -80 °C till use. The disease was staged according to the American Joint Committee on Cancer (AJCC) TNM (tumor–node–metastasis) classification. According to the highly varied survival rate, BCa with stages from I to IIIA having not spread beyond the breast or axillary lymph nodes were named as locoregional BCa, and stages from IIIB to IV as distant BCa in our study. Elderly (≥ 60 years old) patients differed from patients of other ages in terms of prognosis and disease management, so all patients were divided into two categories by 60 years of age. Clinic characteristics of patients were summarized in Table 1.

**Table 1:** Correlation of serum POSTN levels and clinicopathological characteristics of participants

**Characteristics**	**Number (%)**	**POSTN(ng/mL) **Mean ± SD	***P* value**
**Health controls**			
Age (median)	52.1		
> 60	24 (34.8)	11.18 ± 5.51	
≤ 60	45 (65.2)	12.29 ± 11.83	0.30^a^
Menopausal status			
Yes	43 (62.3)	11.79 ± 9.48	
No	26 (37.7)	11.21 ± 5.64	0.63^a^
Family history			
Yes	2 (2.9)	11.57 ± 8.31	
No	67 (97.1)	11.34 ± 3.65	-
Body mass index (BMI)			
< 24	42 (60.9)	11.31 ± 9.62	
≥ 24	27 (39.1)	11.97 ± 5.44	0.18^a^
**Breast cancer**			
Age (median)	50.6		
> 60	50 (28.9)	25.91 ± 16.52	
≤ 60	123 (71.1)	31.18 ± 39.83	0.39^a^
Menopausal status			
Yes	109 (63.0)	29.27 ± 30.35	
No	64 (37.0)	30.31 ± 41.47	0.11^a^
Family history			
Yes	12 (6.9)	25.30 ± 11.50	
No	161 (93.1)	29.98 ± 35.90	0.42^a^
Body mass index (BMI)			
< 24	109 (63.0)	23.04 ± 20.88	
≥ 24	64 (37.0)	40.93 ± 48.44	0.017^a^
TNM stage			
Locoregional BCa (I—IIIA)	115 (66.5)	20.03 ± 18.15	
Distant BCa IV (IIIB—IV)	58 (33.5)	48.73 ± 49.30	< 0.0001^a^
Lymph node metastasis			
Yes	94 (54.3)	36.83 ± 41.78	
No	79 (45.7)	21.12 ± 21.17	0.0002^a^
Distant metastasis			
Yes	36 (20.8)	56.33 ± 54.48	
No	137 (79.2)	22.65 ± 22.95	< 0.0001^a^
Metastasis site			
Multiple sites	17 (47.2)^c^	66.90 ± 61.64	
Single site	19 (52.8)^c^	46.88 ± 46.85	0.24^a^
Bone	10 (27.8)^c^	47.76 ± 58.02	
Lung	5 (13.9)^c^	64.69 ± 35.28	
Liver	3 (8.3)^c^	26.53 ± 8.51	
Brain	1 (2.8)^c^	10.97	
Molecular subtype			
Luminal A	66 (38.2)	30.92 ± 34.17	
Luminal B	60 (34.7)	26.75 ± 27.03	
Her2-enriched	18 (10.4)	37.94 ± 52.33	
Triple negative	29 (16.7)	27.64 ± 37.97	0.81^b^

^a^ Mann–Whitney U test. ^b^ Kruskal–Wallis test. ^c^ Proportion in cases with distant metastasis

Follow-up of patients were performed by interview in the clinic or by telephone every.

4–6 months. Causes of death were assessed by examining medical records. Bone metastasis was assessed by ECT (emission computed tomography). Brain, lung, and liver metastasis were assessed by serial CT scans and MRI. The follow-up time ranged from 6 to 36 months (with mean follow-up time of 26.5 months), and the cutoff date follow-up was 10 April 2021.

### Bioinformatics analysis

TPM (Transcript per million) expression data of BCa in TCGA database were downloaded from UCSC (The University of California Santa Cruz) Xena hubs (http://xena.ucsc.edu/). Differential expression analysis between BCa and non-tumor tissues was performed, and the 250 most significantly up-regulated genes (top up-regulated genes) were obtained; 250 genes with highest TPM values (top abundance genes) in BCa tissues were selected; 908 secreted plasma proteins were predicted by the Human Protein Atlas database. Then, the candidate genes were obtained by taking the intersection of the above gene sets. Finally, to obtain candidates more likely to significantly increase plasma protein levels, their relative abundance in BCa tissues and 21 other different female normal tissues was assessed.

For proteomic analysis, to validate consistent alterations of POSTN protein in BCa, processed expression matrix data (Prot datatype file) was downloaded from Clinical Proteomic Tumor Analysis Consortium (CPTAC, dataset ID: PDC000120), and then, the relative abundance of POSTN in 18 paired BCa and matched non-tumor tissues, as well as other 116 BCa tissues was extracted and compared.

### Measurement of serum POSTN

Soluble POSTN in patients and control subjects were measured by the ELISA method using a commercial kit (R&D System Inc., MN, USA), according to the instructions from the manufacturer, which has been described previously [11]. All measurements were repeated in duplicate.

### Measurement of serum CEA and CA15.3

Serum CA153 and CEA were detected with electrochemiluminescence immunoassays on the Roche Cobas E801 immunoassay analyzer (Roche Diagnostics, Mannheim, Germany) equipped with dedicated reagents, according to the instructions from the manufacturer. All of the assays were performed at the department of Laboratory Medicine, Tianjin Medical University Cancer Institute and Hospital, Tianjin.

### Statistical analysis

Statistical significance was determined with the nonparametric Mann–Whitney U-test (difference in two groups) or Kruskal–Wallis test (difference in more than two groups). Spearman correlation coefficients method was used to evaluate the association between two markers. Receiver operating characteristic (ROC) curves were generated to assess diagnostic efficiency. For combination of biomarkers, binary logistic regression was used to get the probability, which was used as the test variable for ROC curve. The confidence intervals (95% CI) for AUC and *P* value for comparison of related ROC curves were performed by the method described by DeLong and coworkers [12]. Overall and progress survival was analyzed using the Kaplan–Meier product limit method and log-rank test. BCa patients was separated by the median level of POSTN as high and low in all corresponding analysis groups. Proportional hazards models were used to evaluate the association between characteristics of BCa patients and their survival. All of these statistical analyses were performed with SPSS 23.0 software (SPSS Inc., Chicago, IL, USA). Values of *P* ≤ 0.05 were considered statistically significant.

## Results

### Elevated serum levels of soluble POSTN in patients with BCa

First of all, to confirm the up-regulation of POSTN and explore its expression characteristics in BCa tissues, we performed analysis through the Cancer Genome Atlas (TCGA) database and The Human Protein Atlas database. As shown in Supplementary Fig. 1, POSTN is among the 4 genes which are top up-regulated, high abundance and encode secreted plasma proteins in BCa tissues. Then, TPM values of the 4 candidate genes across 21 different female normal tissues and BCa tissues was compared. Only POSTN, a secreted glycoprotein, was observed with low abundance in all normal tissues (Supplementary Fig. 2). Molecular subtype analysis showed that mRNA levels of POSTN were positively associated with the status of ER, PR and Her2. Expression of POSTN was most in molecular subtype of Her2-enriched and least in triple-negative BCa (Supplementary Fig. 3). Finally, proteomic analysis with CPTAC (Clinical Proteomic Tumor Analysis Consortium) mass-spectrometry data [13] further confirmed the up-regulated expression of POSTN protein in BCa tissues, in both paired and unpaired groups (Supplementary Fig. 4). Therefore, we focus the attention on POSTN in the subsequent study.

**Fig. 1:**
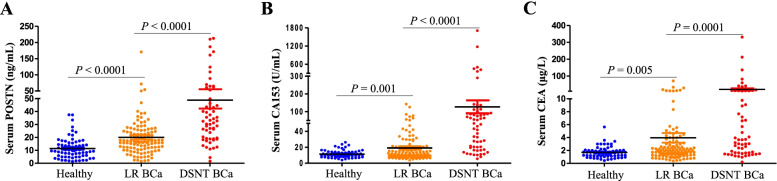
Validation of the elevated serum levels of soluble POSTN in patients with BCa Scatter plots for POSTN, CA153 and CEA in the POSTN (**A**), CA153 (**B**), and CEA (**C**), respectively. For each marker in the dataset, three groups were included: healthy controls (Healthy, *n* = 69), locoregional BCa (LR BCa, *n* = 115) and distant BCa (DSNT BCa, *n* = 58)

Serum levels of POSTN, together with CA153 and CEA were determined in healthy controls and patients with BCa (divided into two groups: locoregional BCa and distant BCa). As shown in Table 1**,** POSTN levels were significantly with BMI (body mass index), TNM stage, lymph node and distant metastasis status in BCa patients, but not with age, menopausal status, and family history. There were no significant differences between patients with different distant metastatic sites. Moreover, in contrast to the results for mRNAs in the TCGA database, no clear correlations were observed between soluble POSTN in serum and the molecular subtypes of ER, PR or Her2 (Supplementary Fig. 5). And as shown in Fig. 1, serum levels of POSTN were significantly increased in patients with locoregional BCa, compared with healthy controls. In addition, POSTN levels in the distant BCa group were also significantly elevated than that in the locoregional BCa group. (Fig. 1A). POSTN showed promising potential to be served as a BCa biomarker. As shown in Table 1, Analogous expression patterns were also observed in CA153 and CEA (Fig. 1B-C).

**Fig. 2:**
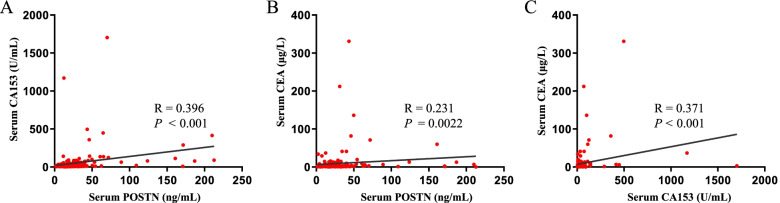
Correlations of serum POSTN CA153 and CEA levels. Using nonparametric Spearman's correlation coefficients method to analyze the correlation between serum POSTN and CA153 (**A**), serum POSTN and CEA (**B**), CA153 and CEA (**C**)

To assess how the serum biomarkers behaved in each group of subjects, we made correlation scatter plots between every two markers. As is illustrated in Fig. 2A-C, no strong correlation was found among the three serum markers, which indicated that POSTN may have complementary roles for CA153 and CEA in the diagnosis of BCa.

### Performances of POSTN in the diagnosis of locoregional BCa

**Fig. 3:**
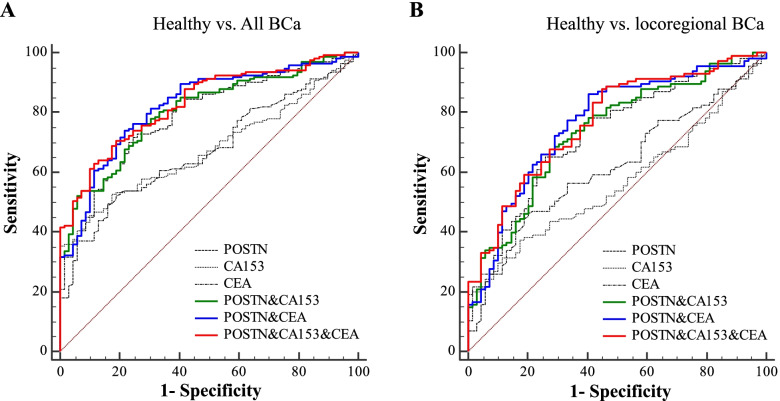
Performances of markers for diagnosis of BCa. ROC curves of POSTN, CA153 and CEA as individual markers or marker panels. **A**, to distinguish all BCa (*n* = 173) from healthy controls (*n* = 69); **B**, to distinguish locoregional BCa (*n* = 115) from healthy controls (*n* = 69). The corresponding subgroups were all indicated above the corresponding ROC curves

As individual biomarkers, performances of POSTN, CA153 and CEA in discriminating all BCa patients from healthy controls were evaluated by the ROC curves, and POSTN was observed with the highest AUCs (AUC_POSTN_ = 0.78 [0.72 – 0.83], AUC_CA153_ = 0.68 [0.61 – 0.74], AUC_CEA_ = 0.68 [0.61 – 0.74], *P* < 0.05, Table 2 and Fig. 3A). Moreover, to distinguish health controls from locoregional BCa, AUCs of POSTN remained the highest among the three markers (AUC_POSTN_ = 0.72 [0.65 – 0.79], AUC_CA153_ = 0.57 [0.49 – 0.64], AUC_CEA_ = 0.62 [0.55 – 0.69], Table 2 and Fig. 3B).

**Table 2:** Performances of biomarkers for the diagnosis of BCa

	**AUC (95% CI)**	**Cut-off value**	**Sensitivity (%)**	**Specificity (%)**
**Healthy vs. All BCa**				
POSTN	0.78 (0.72 – 0.83)^**a1**^	14.5	71.7	75.4
CA153	0.68 (0.61 – 0.74)	16.7	45.7	89.9
CEA	0.68 (0.61 – 0.74)	2.2	49.1	84.0
POSTN + CA153	0.80 (0.75 – 0.85)^**c3**^	-	76.3	72.5
POSTN + CEA	0.82 (0.77 – 0.87)^**c3**^	-	74.0	78.3
POSTN + CA153 + CEA	0.83 (0.78 – 0.87)^**c3**^	-	70.5	81.1
**Healthy vs. locoregional BCa**				
POSTN	0.72 (0.65 – 0.79)^**b**^	14.2	65.2	73.9
CA153	0.57 (0.49 – 0.64)	14.3	37.4	82.6
CEA	0.62 (0.55 – 0.69)	2.1	46.1	79.7
POSTN + CA153	0.74 (0.67 – 0.80)^**c1**^	-	68.7	71.0
POSTN + CEA	0.77 (0.70 – 0.82)^**c3**^	-	86.1	59.4
POSTN + CA153 + CEA	0.77 (0.70 – 0.83)^**c3**^	-	87.8	55.1
**Locoregional BCa vs. distant BCa**				
POSTN	0.78 (0.71 – 0.84)	25.8	67.2	79.1
CA153	0.82 (0.76 – 0.88)	19.3	75.9	74.8
CEA	0.68 (0.61 – 0.75)	2.6	63.8	70.4
POSTN + CA153	0.84 (0.78 – 0.89) ^**3**^	-	84.5	71.3
POSTN + CEA	0.81 (0.74 – 0.86)	-	58.6	92.1
POSTN + CA153 + CEA	0.85 (0.78 – 0.90) ^**3**^	-	74.1	83.5

^**a**^^**−c**^, in comparison with CA153: ^**a**^*, P* < 0.05; ^**b**^, *P* < 0.01; ^***c***^*, P* < 0.001

^**1**^^**–**^^**3**^, in comparison with CEA: ^**1**^*, P* < 0.05; ^**2**^, *P* < 0.01; ^***3***^*, P* < 0.001

To estimate whether the discrimination ability for BCa could be improved by combination among markers, panel models were constructed. As shown in Table 2 and Fig. 3A-B, to discriminate all BCa from healthy controls, both CA153 and CEA were observed with significantly improved AUCs by combination with POSTN (AUC_POSTN + CA153_ = 0.80 [0.75 – 0.85], *P* < 0.001; AUC_POSTN + CEA_ = 0.82 [0.77 – 0.87], *P* < 0.001). And to discriminate locoregional BCa from healthy controls, significant improvements were also observed (AUC_POSTN + CA153_ = 0.74 [0.67 – 0.80], *P* < 0.001; AUC_POSTN + CEA_ = 0.77 [0.70 – 0.82], *P* < 0.001).

### Performances of POSTN in predicting distant metastasis of BCa

**Fig. 4:**
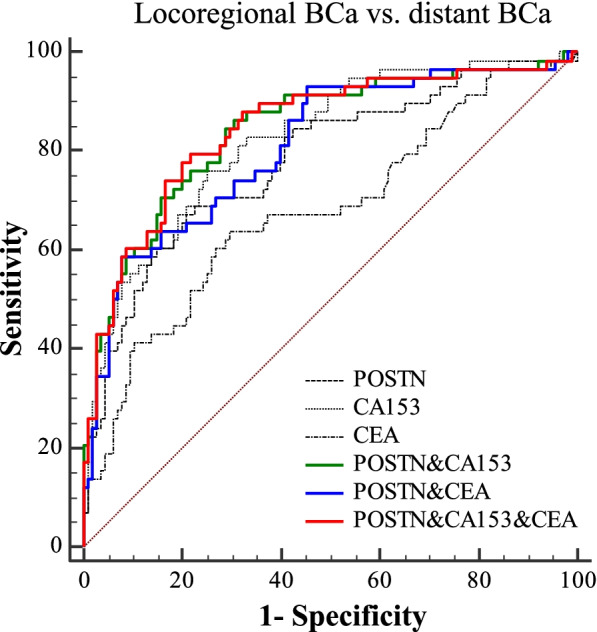
Performances of markers for metastasis prediction of BCa. ROC curves of POSTN, CA153 and CEA as individual markers or marker panels. To distinguish distant BCa (*n* = 58) from locoregional BCa (*n* = 115). The corresponding subgroups were indicated above the corresponding ROC curves

Next, the potential of POSTN in predicting distant metastasis of BCa was evaluated. To distinguish distant BCa from locoregional BCa, the performances of the POSTN and CA153 were comparable (AUC_POSTN_ = 0.78 [0.71 – 0.84], AUC_CA153_ = 0.82 [0.76 – 0.88], Table 2 and Fig. 4). Moreover, the AUC three marker panel composed of POSTN, CA153 and CEA was higher than that of CA153 and CEA alone (AUC_POSTN +CA153 + CEA_ = 0.85 [0.78 – 0.90]).

### Performances of POSTN in diagnosis of CA153 or CEA-negative BCa patients

**Fig. 5:**
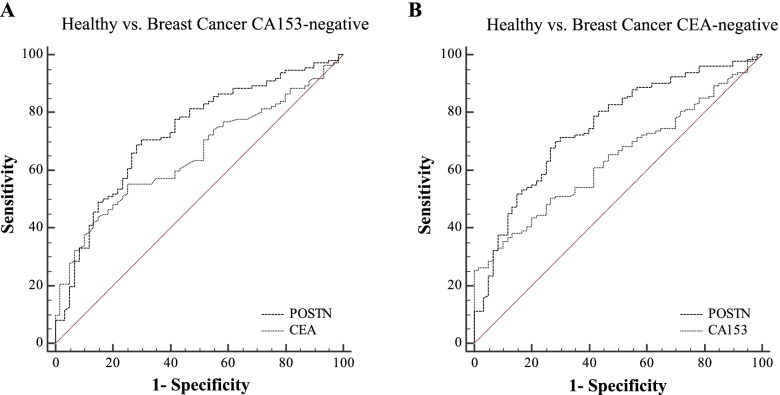
Performances of POSTN in diagnosis of CA153 or CEA-negative BCa patients ROC curves of POSTN as individual markers to distinguish CA153-negative (**A**, *n* = 112) or CEA-negative (**B**, *n* = 134) BCa patients from healthy controls (*n* = 69), respectively. The corresponding subgroups were all indicated above the corresponding ROC curves

CA153 and CEA are the most commonly used serum markers for BCa management. However, if determined by their clinically used cut-off values (CA153, 25 U/mL; CEA, 5 μg/L), as much as 112 (64.7%) and 134 (77.5%) patients would be missed by those markers. In order to further investigate the complementarity of POSTN for CA153 and CEA in the diagnosis of BCa, we assessed its performances for BCa patients which were missed by CA153 or CEA, based on the clinically used threshold. POSTN retained significant ability to discriminate healthy controls from CA153-negative BCa (AUC_POSTN_ = 0.72 [0.65 – 0.79], AUC_CEA_ = 0.65 [0.57 – 0.72]), or CEA-negative BCa (AUC_POSTN_ = 0.75 [0.68 – 0.81], AUC_CA153_ = 0.63 [0.56 – 0.70]), as shown in Table 3 and Fig. 5A-B.

**Table 3:** Performances of POSTN in diagnosis of CA153-negative and CEA- negative BCa patients

Analysis groups	AUC (95% CI)	Cut-off value	Sensitivity (%)	Specificity (%)
**Healthy vs. BCa CA153-negative**				
POSTN	0.72 (0.65 – 0.79)	14.2	70.5	70.0
CEA	0.65 (0.57 – 0.72)	1.8	55.3	75.0
**Healthy vs. BCa CEA-negative**				
POSTN	0.75 (0.68 – 0.81)	14.5	70.1	71.7
CA153	0.63 (0.56 – 0.70)	20.3	32.3	93.3

### Prognostic value of POSTN in patients with BCa

**Fig. 6:**
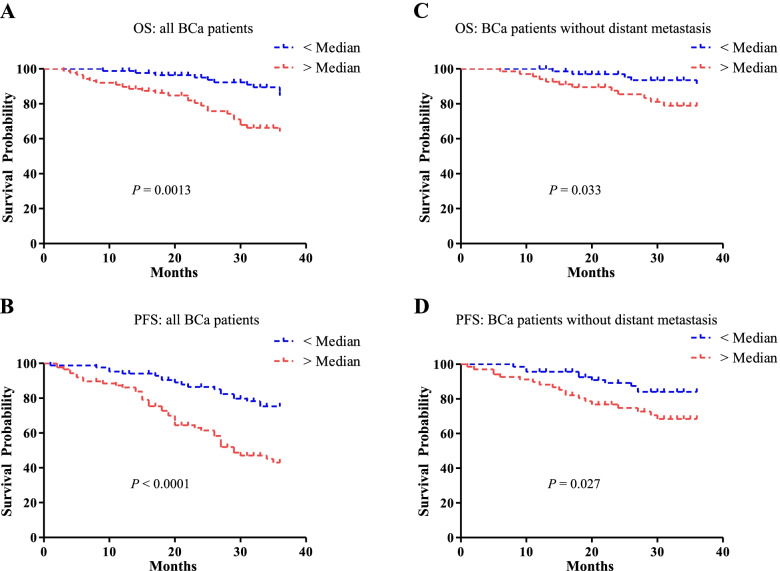
Prognostic value of POSTN in patients with BCa Kaplan–Meier analysis were performed in overall survival (**A** and **C**) and progression-free survival (**B** and **D**) between high POSTN group (> Median, *n* = 86) and low POSTN group (< Median, *n* = 87). Survival analysis without (**C** and **D**) distant metastases were also taken into consideration. Statistical significance was determined with log-rank test

To determine the prognostic value of serum POSTN in BCa, the relationship between the serum POSTN and clinical outcome were analyzed. Statistically significant difference in overall survival and progression-free survival was observed between high POSTN group and low POSTN group (Fig. 6A-B, *P* = 0.0013 and *P* < 0.0001, respectively). Considering that patients with distant metastases account for the majority of overall death and progression, survival analysis without those patients were also performed, and consistent results were obtained (Fig. 6C-D, *P* = 0.033 and *P* = 0.027, respectively). Patients with high levels of POSTN tended to have shorter overall and progression-free survival time. Proportional hazards models were used to evaluate the association between POSTN as well as other characteristics of BCa patients and survival (as shown in Supplementary Table 1). Multivariate analysis including variables such as TNM stage and metastasis status indicated that soluble POSTN was no independent prognostic factor for BCa.

## Discussion

The limitations of the current use of imaging for BCa screening are high costs, time-consuming, side effects from exposure to radiation and contrast agents[7]. The most commonly used blood markers in BCa management, CA153 and CEA, provide incompetent performances due to limited sensitivity and specificity [14]. In the present study, we developed a pipeline to integrate the transcriptome and plasma proteome to explore circulating protein biomarkers for BCa, and focused on POSTN. Soluble POSTN were observed with significantly up-regulated expression in patients with BCa, and was provided with promising value in early diagnosis, distant metastasis prediction and prognostic judgment. Taken together, the current study suggest that soluble POSTN is an informative serum biomarker for BCa.

Recent years have witnessed the dramatically development of mass spectrometry (MS)-based proteomics, which could measure fragmentation spectra of peptides derived from proteins with high-precision [15]. And application of MS-based plasma proteomics in the discovery of plasma biomarkers has been advocated [15]. However, it requires technologies with higher sensitivity and throughput than those available today [16]. Therefore, none of the plasma proteins identified based on mass spectrometry have been used in clinical practice so far. Nevertheless, comprehensive analysis of the proteome results and other "omics" data through bioinformatics methods may provide enormous help in discovering novel blood biomarkers. We developed a pipeline by integrating the transcriptome data from TCGA database and predicted plasma proteome data from The Human Protein Atlas database, the primary purpose of which was to discover secreted plasma protein-coding genes with top up-regulation and abundance at the transcriptional level in BCa tissues. The present study provided a novel strategy for circulating protein marker discovery in malignant disease, and not limited to BCa.

POSTN is a secreted extracellular matrix (ECM) glycoprotein, which was originally identified in osteoblasts [17]. Overexpression of POSTN have been implicated in several types of human cancers [18]. In BCa, immunohistochemistry results indicated that POSTN was overexpressed mainly in cancer-associated stroma and/or slightly in cancer cells, usually associated with poor clinical outcomes [19–22]. Moreover, there was a trend of increasing POSTN expression in metastatic lymph nodes, as well as other tissues and organs [20, 22]. And recent studies have indicated that POSTN played essential roles in numerous malignancies [18]. As for BCa, it was reported that POSTN participated in the process of BCa cell motility and invasion [20, 23, 24], the maintenance of BCa stem cells [25], and the induction of chemoresistance [26, 27]. However, except for limited reports related to prognosis [28–30], few studies have focused on the clinical significance of serum POSTN in BCa patients. In this study, serum POSTN levels were found to be highly elevated in patients with BCa, which were consistent with previous studies reporting increased levels of POSTN mRNA in BCa tissues [22].

To our knowledge, this is the first study that comprehensively evaluated the clinical significance of soluble POSTN in early diagnosis, metastasis prediction, and prognosis of BCa patients. Similar to CEA and CA153, serum POSTN was not elevated in all BCa patients, and there were obvious individual differences in serum POSTN levels, depending on tumor metastatic status, volume of the tumors, subtypes of tumor lesions and specific microenvironment of tumor cell. Our results indicated that, to distinguish health controls from locoregional BCa, POSTN was observed with the highest AUC, and both CA153 and CEA were observed with significantly improved AUCs by combination with POSTN. Moreover, the performances of the POSTN were comparable with that of CA153 in predicting distant metastasis of BCa. Previous published studies suggested that CEA and CA15.3 performed with low sensitivity and specificity, especially with respect to their applications in diagnosing BCa in the early phase [31], but may be useful biomarkers in the management of advanced BCa [9]. Although all classified as secreted glycoprotein, the primary difference between POSTN and traditional BCa biomarker, such as CA153 and CEA, lay in the fact that the former in blood stream was mainly originated from the ECM [19–22], and the latter were are antigenic materials on the surface of or secreted by tumor cells. Compared with normal matrix, POSTN was especially highly up-regulated in cancer-associated stroma cells [19–22], indicating that signals derived from tumor cells, TGF-β, for instance, may be the main inducers of POSTN expression in the cancer-associated ECM [32–36].

Limitations of this study should also to be noted. The median follow-up time of this study was relatively short for BCa patients, which led to the fact that patients with distant metastases account for the majority of overall death and progression. Although overall survival and progression free survival analysis suggested that POSTN possessed prognosis value in BCa patients, soluble POSTN was no independent prognostic factor for BCa. In time-to-event analysis, sufficient follow-up time to capture enough events is the key element to have adequate statistical power. Therefore extended follow-up is ongoing to fully assess the prognosis value of POSTN for BCa patients. The present study was also limited by the relatively small sample size. On the one hand, differences between markers to distinguish locoregional BCa from distant BCa, for instance, failed to reach statistical significance. On the other hand, there were substantial differences in overall survival (OS) and progression-free survival (PFS) according to tumour subtypes of BCa, but the limited sample size makes it difficult to perform subdivision in the molecular types of BCa. And it would be interesting to explore the different clinical significance of soluble POSTN in various BCa subtypes.

## Conclusion

The present study showed that soluble POSTN was a promising biomarker for BCa patients. Performing soluble POSTN evaluation for early detection and metastasis prediction may be beneficial to patients with BCa. Although this work was focused on its potential on early detection and metastasis prediction in BCa, we anticipate that soluble POSTN could also find more clinical implications in the management of BCa, such as chemotherapy efficacy monitoring.

## Supplementary Information

**Additional file 1. **Supplementary figures 1-5

**Additional file 2.** Supplementary table 1

## Data Availability

The datasets analyzed during the current study are available in the [UCSC Xena hubs] repository, [TCGA Breast Cancer cohort]; and [CPTAC] repository, [https://cptac-data-portal.georgetown.edu/study-summary/S039, PDC000120, Prospective Breast BI Proteome]. The datasets used during the current study are available from the corresponding author on reasonable request.

## Abbreviations

AJCC: American Joint Committee on Cancer
AUC: Area under curve
BCa: Breast cancer
BMI: Body mass index
CA153: Carbohydrate antigen 153
CEA: Carcinoembryonic antigen
CPTAC: Clinical Proteomic Tumor Analysis Consortium
CT: Computerized tomography
DSNT BCa: Distant BCa
ECM: Extracellular matrix
ECT: Emission Computed Tomography
ELISA: Enzyme linked immunosorbent assay
ER: Estrogen receptor
Her2: Human epidermal growth factor receptor 2
LR BCa: Locoregional BCa
MRI: Magnetic resonance imaging
MS: Mass spectrometry
OS: Overall survival
PFS: Progression-free survival
POSTN: Periostin
PR: Progesterone receptor
ROC: Receiver operating characteristic
TCGA: Cancer Genome Atlas
TNM: Tumor node metastasis
TPM: Transcript per million
UCSC: The University of California Santa Cruz
