# Supplementary material for: Soluble POSTN is a novel biomarker complementing CA153 and CEA for breast cancer diagnosis and metastasis prediction
Source: BMC Cancer. 2022 Jul 12;22:760. doi: 10.1186/s12885-022-09864-y (PMC9281047; doi:10.1186/s12885-022-09864-y)

Supplementary Figure 1

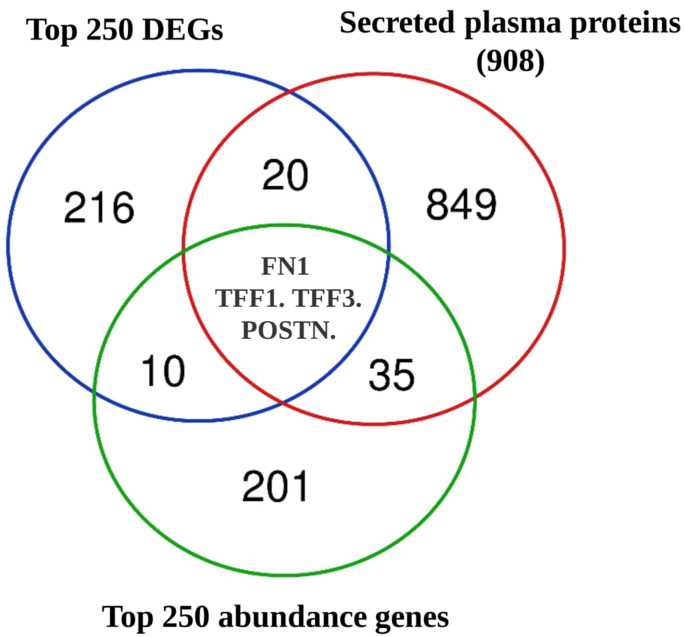

Supplementary Figure 2

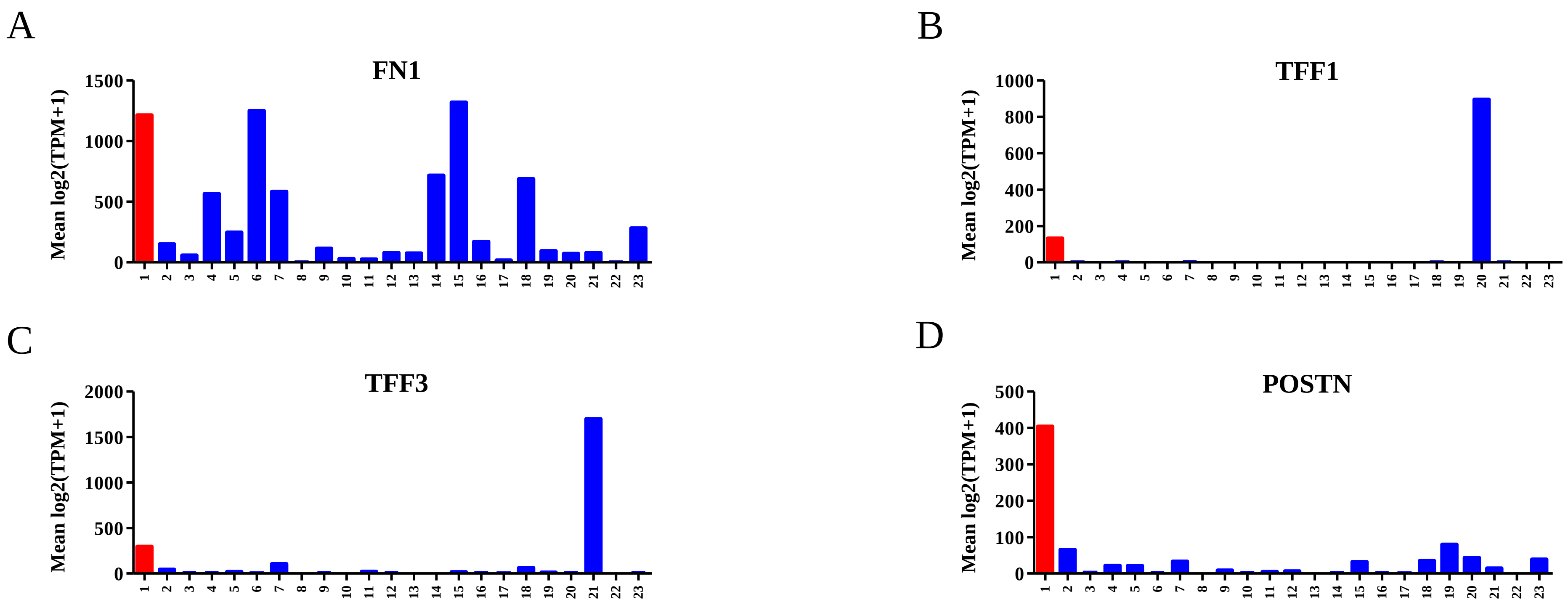

Supplementary Figure 3

A

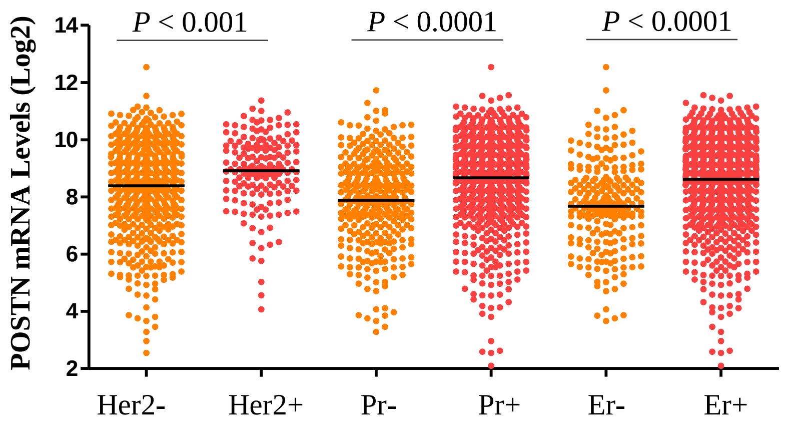

B

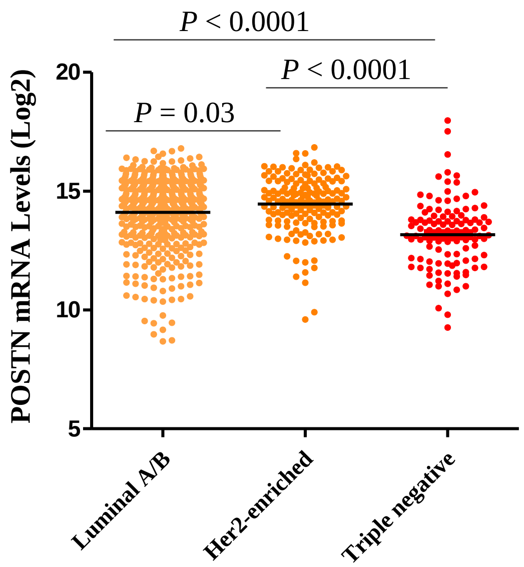

Supplementary Figure 4

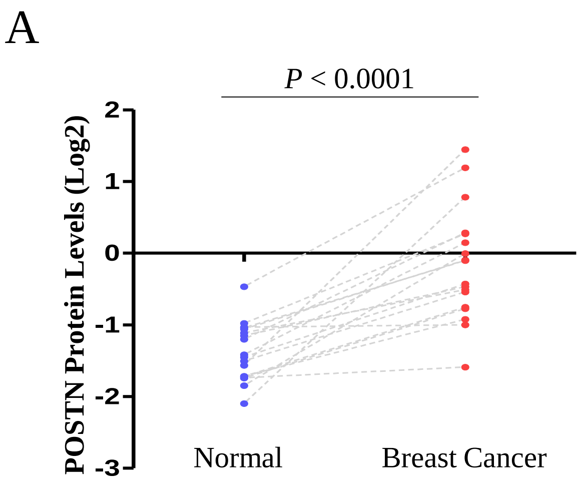

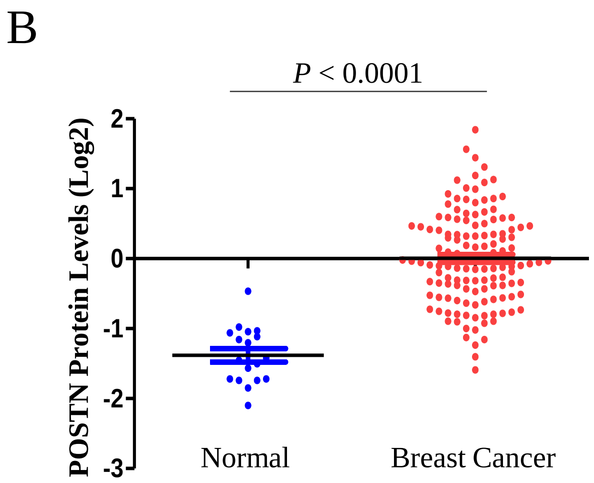

Supplementary Figure 5

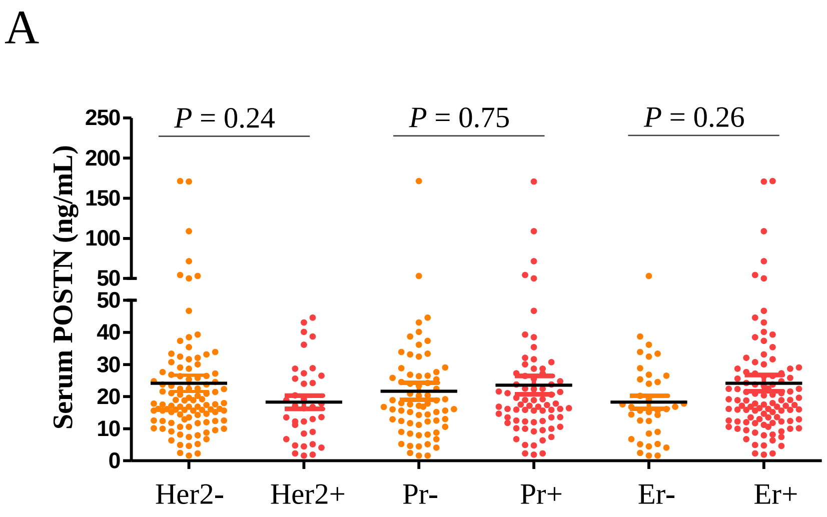

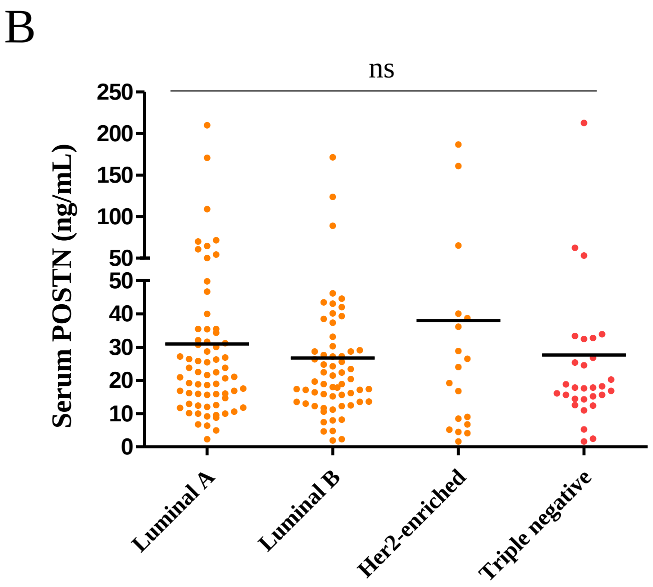

Supplement: Supplementary file 1 — Additional file 1. Supplementary figures 1-5 [file 12885_2022_9864_MOESM1_ESM.pdf]
