# Supplementary material for: Soluble POSTN is a novel biomarker complementing CA153 and CEA for breast cancer diagnosis and metastasis prediction
Source: BMC Cancer. 2022 Jul 12;22:760. doi: 10.1186/s12885-022-09864-y (PMC9281047; doi:10.1186/s12885-022-09864-y)
Supplement: Supplementary file 2 — Additional file 2. Supplementary table 1 [file 12885_2022_9864_MOESM2_ESM.docx]

**Supplementary Table 1. Proportional hazards model to evaluate the association between survival and characteristics of BCa patients.**

| **Progress parameters** | **Univariable analysis** | | **Multivariable analysis** | |
| --- | --- | --- | --- | --- |
| **OS** | **HR (95% CI)** | ***P* value** | **HR (95% CI)** | ***P* value** |
| Age | 1.01 (0.98 – 1.04) | 0.58 | - | - |
| Menopausal status (**+** vs. -) | 1.17 (0.59 – 2.32) | 0.66 | - | - |
| Stage | 2.31 (1.64 – 3.25) | <0.001 | 1.32 (0.50 – 3.47) | 0.57 |
| Lymph node metastasis (**+** vs. -) | 5.38 (2.24 – 2.90) | <0.001 | 2.34 (0.61 – 9.00) | 0.22 |
| Distant metastasis (**+** vs. -) | 4.91 (2.57 – 9.39) | <0.001 | 1.66 (0.37 – 7.53) | 0.51 |
| PR status (**+** vs. -) | 1.66 (0.85 – 3.23) | 0.14 | - | - |
| ER status (**+** vs. -) | 1.42 (0.65 – 3.10) | 0.38 | - | - |
| HER2 status (**+** vs. -) | 1.05 (0.47 – 2.12) | 0.91 | - | - |
| Molecular subtypes |  |  |  |  |
| Luminal A | 1 |  | - | - |
| Luminal B | 0.95 (0.36 – 2.51) | 0.92 | - | - |
| Her2-enriched | 1.43 (0.56 – 3.64) | 0.46 | - | - |
| Triple negative | 0.54 (0.11 – 2.67) | 0.45 | - | - |
| POSTN | 1.11 (1.00 – 1.22) | 0.001 | 1.01 (0.99 – 1.02) | 0.15 |
| **PFS** | **HR (95% CI)** | ***P* value** | **HR (95% CI)** | ***P* value** |
| Age | 1.01 (0.98 – 1.03) | 0.66 | - | - |
| Menopausal status (**+** vs. -) | 1.48 (0.85 – 2.55) | 0.17 | - | - |
| Stage | 2.63 (2.01 – 3.44) | <0.001 | 1.83 (0.84 – 4.01) | 0.13 |
| Lymph node metastasis (**+** vs. -) | 5.00 (2.65 – 9.40) | <0.001 | 1.45 (0.51 – 4.12) | 0.49 |
| Distant metastasis (**+** vs. -) | 6.49 (3.90 – 10.80) | <0.001 | 1.65 (0.50 – 5.46) | 0.41 |
| PR status (**+** vs. -) | 1.29 (0.78 – 2.14) | 0.31 | - | - |
| ER status (**+** vs. -) | 1.24 (0.69 – 2.22) | 0.47 | - | - |
| HER2 status (**+** vs. -) | 1.10 (0.62 – 1.94) | 0.75 | - | - |
| Molecular subtypes |  |  |  |  |
| Luminal A | 1 |  | - | - |
| Luminal B | 1.06 (0.51 – 2.23) | 0.92 | - | - |
| Her2-enriched | 1.19 (0.57 – 2.51) | 0.46 | - | - |
| Triple negative | 0.76 (0.26 – 2.23) | 0.45 | - | - |
| POSTN | 1.01 (1.00 – 1.02) | 0.001 | 1.00 (0.99 – 1.02) | 0.72 |
